# Uterine Leiomyoma in the Context of Uterine Didelphys: A Case Report

**DOI:** 10.7759/cureus.44791

**Published:** 2023-09-06

**Authors:** Asilis J Defran, Chancée Forestier, Ene Morgan, Michelle Thomas

**Affiliations:** 1 General Surgery, Ascension Providence Hospital, Southfield, USA; 2 Obstetrics and Gynecology, Ascension Providence Hospital, Southfield, USA

**Keywords:** medical management of leiomyomas, surgical management of leiomyomas, uterine anomalies, uterine didelphys, leiomyomas

## Abstract

Uterine leiomyomas are one of the most common reproductive pathologies in born females. The majority of women within reproductive age will develop a leiomyoma, most of which will be asymptomatic. Though there has been extensive research regarding this pathology alone, there is more to be learned about leiomyomas that affect women with other comorbidities. This case study reviews the medical and surgical management of a woman born with two uteri, medically termed congenital uterus didelphys. Within her reproductive years, she develops symptomatic leiomyomas in each of her uteri and seeks surgical management. This case study aims to widen the scientific knowledge surrounding these subsets of women with a common diagnosis superimposed on an extremely rare diagnosis.

## Introduction

Uterine leiomyomas, colloquially known as fibroids, have an estimated presence of 77% among women of reproductive age [1]. Congenital uterine anomalies exist in approximately 4.3% of fertile women [2]. Of the 4.3% of patients, uterine didelphys is present in only 8% [3]. Given these figures, a case possessing these features has a 0.0026% chance of occurrence. Therefore, when the patient presented with leiomyomas in each of her uteri, it was crucial to share this case due to its rarity. 

Uterine leiomyomas are benign monoclonal tumors originating from the myometrium's smooth muscle cells and fibroblasts [4]. Histologically, leiomyomas appear benign lesions of smooth muscle cells with large amounts of interspersed extracellular matrix; this amalgamation of cells is surrounded by a thin pseudocapsule of areolar tissue and compressed muscle fibers [5]. Following a review of the pathogenesis, symptomatology, and treatment of leiomyomas, we will present the case and surgical management of a 32-year-old African-American female with uterus didelphys and multiple symptomatic leiomyomas.

Pathogenesis of leiomyomas

Although the pathogenesis of leiomyomas has yet to be fully uncovered, studies demonstrate consistent findings of karyotype abnormalities in 40% to 50% of patients, including translocations (HMGA2, RAD51B) and deletions (7q). In these cases, chromothripsis, defined as events resulting in 20 or more intrachromosomal breakpoints, is proposed as the initiating event that provokes tumorigenesis in uterine smooth muscle cells. Point mutations (MED12) and enzyme deficiencies (Fumarate hydratase) have also been implicated in developing leiomyomas in karyotypically normal smooth muscle cells. However, an important tumor suppressor gene, P53/TP53, remained unaffected, and gene amplification was not discovered in this gene; these factors may play a role in preventing the transition into a malignant nodule [1].

Symptomatology of leiomyomas

Fibroids are often subject to hormonal influence. Symptomatic patients generally experience the greatest exacerbations during menses, as they are largely affected by the fluctuations of estrogen and progesterone levels. Relative to normal myometrium, the leiomyoma cells have increased steroid and growth hormone receptors, which are estrogen-regulated elements. Leiomyomas attain higher tissue concentrations of estrogen relative to normal myometrium due to their higher concentrations of aromatase. Though the causative relationship between progesterone and leiomyomas is still under investigation, the current research demonstrates a positive correlation between progesterone levels and leiomyoma growth rate. It has been shown that myometrial bcl-2, an antiapoptotic gene, reaches its highest level of activity during the progesterone-rich secretory phase of menses. In postmenopausal women, symptoms tend to wane in severity, and the fibroids decrease in size due to the diminished hormonal availability [6]. These hormonal influences help illuminate why leiomyomas are most often found in women of reproductive age. 

Uterine leiomyomas are clinically apparent in 25% of women [7]. Classic symptomatology of uterine leiomyomas includes heavy or prolonged vaginal bleeding during menstruation, pelvic pressure or pain, and increased urinary frequency or feelings of incomplete voiding. Secondary manifestations of symptomatic leiomyomas to consider are hydronephrosis due to ureter compression, bowel obstruction from mass effect, and thromboembolisms due to venous stasis [4]. Patients may also experience infertility or adverse obstetric outcomes such as placenta abruptio or malpresentation. The different locations of fibroids can be more likely than others to cause certain symptomatology. For example, submucosal and intramural leiomyomas are the most likely to cause infertility [4].

Treatment of leiomyomas

Treatment options for uterine leiomyomas include both medical and surgical modalities. Medication management of leiomyomas is largely targeted toward reducing the severity of symptoms as opposed to being curative. Combination oral contraceptives are a common first-line agent for symptomatic control of menorrhagia before definitive treatment; however, there is not enough evidence to prove this approach is effective [7]. However, it stands to reason that the anovulatory cycles produced by low-dose combination OCPs may be detrimental to leiomyoma growth. Progestin-releasing intrauterine devices have also been used to treat heavy menses associated with fibroids. Gonadotropin-releasing hormone (GnRH) agonists are most effective at targeting the range of symptomatic leiomyomas because they have been shown to reduce the uterine size by 35% to 65%, relieving pelvic pain or mass effect symptoms and may produce amenorrhea [7]. Though these size-reducing effects rapidly reverse upon discontinuation of the medication, GnRH agonists are primarily used to reduce fibroid size and symptoms before surgery or to transition a patient to menopause [8]. Surgical management of leiomyomas aims to resolve symptoms by removing or destroying the offending agent. The procedure of choice depends on several factors, including the patient's desire for future fertility, the desire to retain the uterus, and the FIGO leiomyoma type. In patients with FIGO type 0, 1, and 2 who have aimed for future fertility, hysteroscopic myomectomy is the procedure of choice [9]. In patients with fibroids not amenable to a transcervical approach, the recommendation is to perform a laparoscopic or open abdominal procedure, depending on fibroid size. Additional procedures to consider are MRI-guided focused ultrasound surgery, uterine artery embolization, radiofrequency ablation, or hysterectomy [8]. Despite surgical treatment, leiomyomas may still recur due to remnants of a previously removed nodule taking root or an entirely new fibroid emerging; due to the risk of recurrence, hysterectomy remains the only definitive procedure to relieve symptoms and prevent future occurrences.

## Case presentation

**Figure 1:**
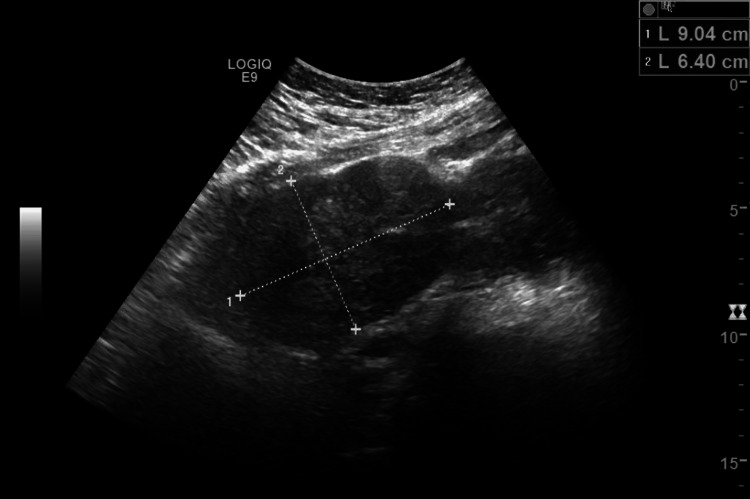
Initial imagining demonstrating the patient's right uterus.

**Figure 2:**
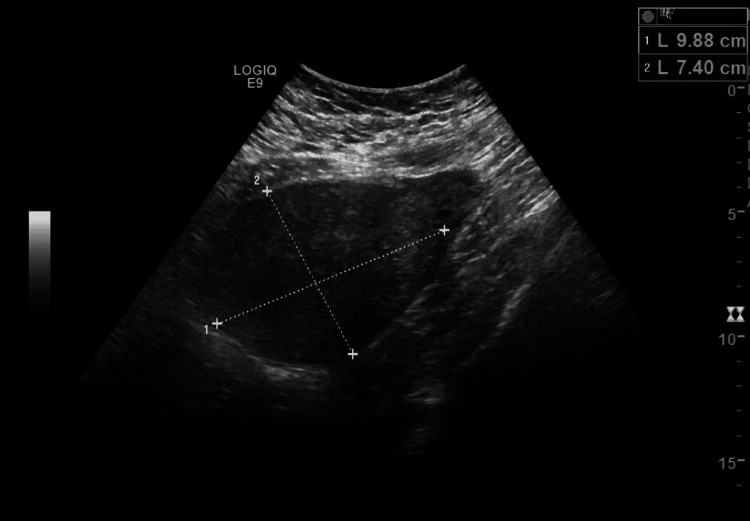
Initial image demonstrating the patient's left uterus.

A 32-year-old African-American female with no medical history initially presented with vaginal bleeding during her first pregnancy. Radiology confirmed the presence of two uteri, with the right uterus measuring 9.0 x 6.4 x 6.2 cm (Figure 1), the left uterus measuring 9.9 x 7.4 x 8.0 cm (Figure 2), and a 0.8 cm left intrauterine pregnancy. Additionally, multiple fibroids on the superior aspect of both uteri were noted. This pregnancy would later end in an elective abortion.

**Figure 3:**
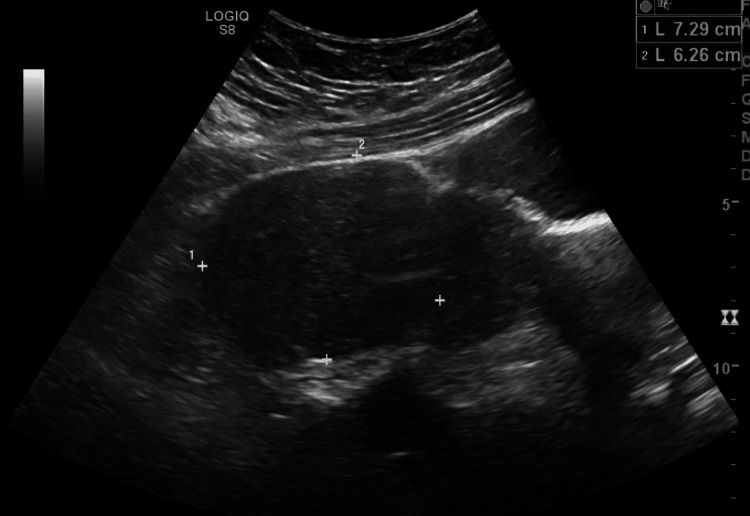
Right uterine subserosal right lateral fibroid.

Eleven months later, the patient was seen for concerns of heavy vaginal bleeding. Pelvic ultrasound with supplementary transvaginal scanning demonstrated a 7.3 x 6.3 x 7.5 cm subserosal right lateral uterine fibroid arising from the right uterus, FIGO 6, (Figure 3) and a 7.1 x 6.2 x 7.7 cm subserosal/wide-based pedunculated fundal fibroid arising from the left uterus, FIGO 7. The endometrial stripes of both uteri were normal. The patient did not want further management at this time.

**Figure 4:**
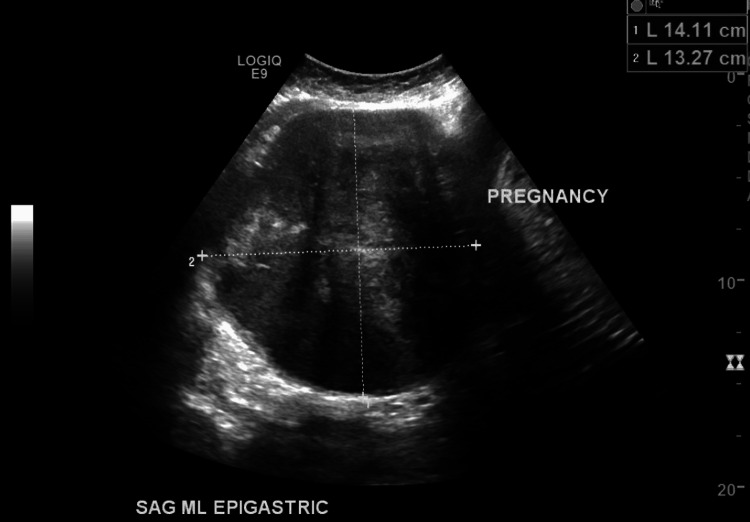
Subserosal fibroid visualized on the gravid uterus.

The patient would be lost to follow-up until she presented with abdominal pain the following year at 15 weeks gestation. At 27 weeks gestation, a subserosal, FIGO 6, fibroid visualized on the gravid uterus measured 14.3 x 14.1 x 13.3 cm (Figure 4). The patient experienced prelabor rupture of membranes, followed by an arrest of labor, and underwent an at-term cesarean section. The left uterine fibroid previously seen on imaging was visualized during the procedure, and two smaller fundal fibroids were palpated on the left gravid uterus. After delivery, the patient's uterus could not be exteriorized for repair of the low transverse section due to fibroid size and was wrapped in a damp towel so that any remaining products of conception could be cleared. The uterine incision was closed in one layer of polysorb suture, and the patient was closed once excellent hemostasis was noted.

**Figure 5:**
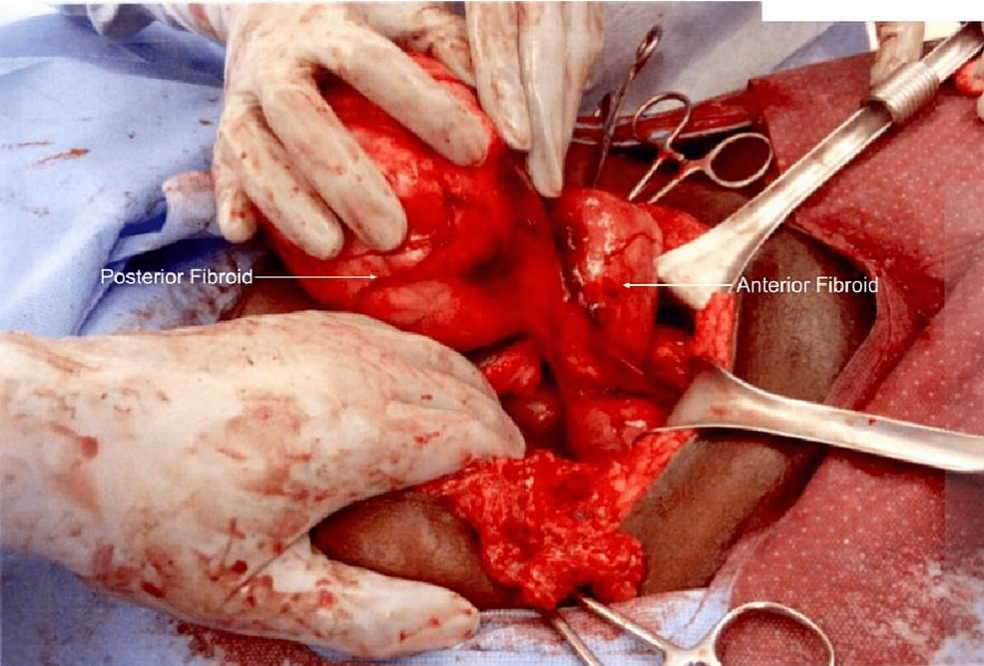
Anterior and posterior left uterine fibroids.

**Figure 6:**
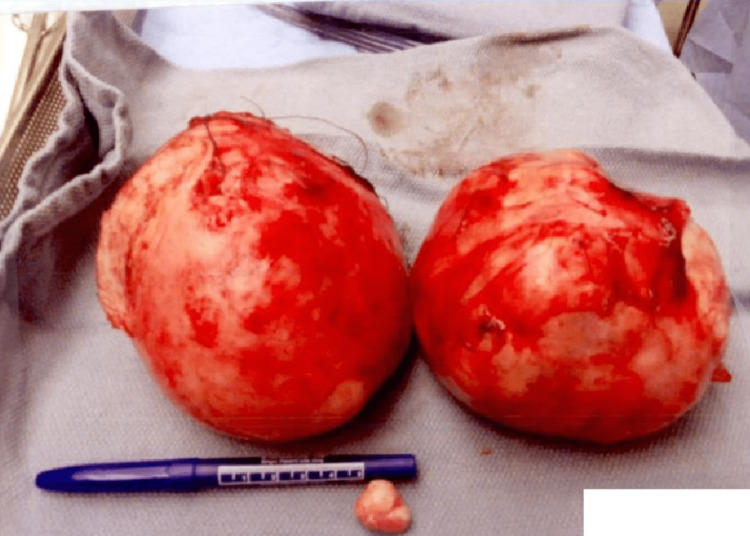
Two large fibroids from the left uterus. Small fibroid from the right uterus.

She presented for a scheduled myomectomy pre-operative interview four months after the cesarean delivery. The patient expressed continued fibroid-associated abdominal and pelvic pain exacerbated by menstruation. During the myomectomy procedure, there were two large pedunculated fibroids, measuring about 14 cm, arising from the fundus of the left horn of the didelphys uterus. One is located posteriorly, and the other anteriorly (Figure 5). A small 3 cm subserosal fibroid was also arising from the right horn of the didelphys uterus. The left posterior fibroid was removed first after injection with vasopressin to its stalk and then resection using electrocautery. The anterior fibroid had a softer consistency, consistent with fat necrosis. The stalk was identified, infused with vasopressin, and then transected. The small leiomyoma found in the right fundal horn was removed with gentle traction and electrocautery. Figure 6 displays the three resected leiomyomas. Excellent hemostasis was confirmed at the end of the procedure, and the patient was closed with an estimated blood loss of 700 mL, operative time of 2 hours and 52 minutes. The postoperative course was uncomplicated, and she was discharged home after three days with instructions to follow up in two weeks.

## Discussion

The patient's course and imaging highlight the volatile nature of leiomyomas and the radiologic difficulty of tracking leiomyomas in the context of uterine didelphys. To shed more light on the patient's anatomy, we will discuss the genesis of congenital uterine anomalies: From week 6 to 12 of female fetal development, caudal elongation of both mullerian ducts occurs, allowing for a fusion of the two ducts at the midline and insertion into the dorsal urogenital sinus. This sets the stage for development of the fallopian tubes, uterus, cervix, and upper third of the vagina. To complete the process, canalization of the two fused ducts and absorption of the intervening septum in the cephalad direction concludes by week 20. Most anomalous uterine presentations, such as septate, bicornuate, unicornuate, and didelphys, develop secondary to difficulties during elongation, fusion, canalization, or absorption of the intervening septum. The different congenital uterine anomalies have been classified and revised in 2021 by the American Society of Reproductive Medicine [10]. In uterine didelphys, failure of the fusion between the two Mullerian ducts results in individual insertions into the urogenital sinus. Further development of the two independent mullerian ducts leads to forming two distinct uteri and cervixes. Notable complications of uterine didelphys are spontaneous abortions in 32% and preterm births in 28% [3].

The cornerstone of classification presented by the International Federation of Gynecology and Obstetrics standards, Table 1, proved invaluable in properly identifying each fibroid and thus planning the optimal surgical approach in light of the leiomyoma's unforeseeable developments.

**Table 1:** International Federation of Gynecology and Obstetrics classifications of Leiomyomas.

Type	Description
0	Submucosal, pedunculated
1	Submucosal, less than 50% intramural
2	Submucosal, greater than 50% intramural
3	Intramural but contacting endometrium
4	Intramural
5	Subserosal, greater than 50% intramural
6	Subserosal, less than 50% intramural
7	Subserosal, pedunculated
8	Non-myometrial location (ex. Cervical, broad ligament…)

After discovery, the subserosal/wide-based pedunculated fundal fibroid arising from the left uterus, initially measuring 7.1 x 6.2 x 7.7 cm, nearly doubled in size to a measurement of 14.3 x 14.1 x 13.3 cm over 13.5 months, under the hormonal influences of pregnancy. The left uterine fibroid would then decrease in size to a measurement of 14 x 13 x 11.6 cm over six months. During the myomectomy, two fibroids were ultimately excised from the left uterine horn, each approximately 14 cm.

The initially imaged subserosal right lateral leiomyoma arising from the right uterus measuring 7.3 x 6.3 x 7.5 cm had a less detailed and, subsequently, more difficult course to follow. The myomectomy ended with the excision of a 3 cm subserosal fibroid from the right uterine horn. It is possible that the leiomyoma shrunk in size or that the initially imaged subserosal right lateral leiomyoma was mistaken for a leiomyoma arising from the left uterus.

The patient's presentation of worsening abdominal pain leading up to the myomectomy, debris evacuated from one of the fibroids intraoperatively, and the surgical pathology report of infarct type necrosis supports post-pregnancy fibroid degeneration as the underlying progression. The patient's leiomyomas greatly grew during the months of her second pregnancy. The postpartum effects on uterine vasculature and patient hormones created an environment that could no longer support fibroids of that size, which led them to necrotize and degenerate.

## Conclusions

Uterine leiomyomas are benign proliferating nodules of smooth muscle cells. Symptomatic patients tend to be of reproductive age, presenting with symptoms of heavy or prolonged vaginal bleeding, pelvic pain, increased urinary frequency, constipation, or infertility. In the context of a congenital uterine anomaly, uterine didelphys, in this case, management of leiomyomas can prove challenging due to the deviation from normal anatomy and difficulties in distinguishing imaging. Surgical management can include a myomectomy for women who prefer to keep their fertility status or a hysterectomy for women not interested in future pregnancies. This patient was born with uterine didelphys, which was diagnosed simultaneously with her leiomyomas during her first pregnancy when she experienced vaginal bleeding. She refused medical or surgical management until her second pregnancy, when she re-experienced bleeding and pain. After the Post-cesarean section, she underwent a myomectomy to keep her fertility status. Remarkably, the patient's leiomyomas changed drastically in size throughout her course, possibly due to the hormonal changes from her pregnancies. Each patient's pathology, uterine location, comorbidities, and preferences must be considered to find the best management for the patient's treatment goals.
